# An Australian rental housing conditions research infrastructure

**DOI:** 10.1038/s41597-022-01136-5

**Published:** 2022-02-02

**Authors:** Emma Baker, Lyrian Daniel, Andrew Beer, Rebecca Bentley, Steven Rowley, Michelle Baddeley, Kerry London, Wendy Stone, Christian Nygaard, Kath Hulse, Anthony Lockwood

**Affiliations:** 1 Australian Centre for Housing Research, The University of Adelaide, Adelaide, 5005 Australia; 2 UniSA Business, The University of South Australia, Adelaide, 5000 Australia; 3 Melbourne School of Population and Global Health, The University of Melbourne, Melbourne, 3000 Australia; 4 School of Accounting, Economics and Finance, Curtin University, Pert, 6000 Australia; 5 UTS Business School, University Technology Sydney, Sydney, 2007 Australia; 6 Office of the Pro Vice Chancellor Research, Torrens University Australia, Sydney, 2007 Australia; 7 School of Arts, Social Sciences and Humanities, Swinburne University of Technology, Melbourne, 3122 Australia

**Keywords:** Sociology, Geography

## Abstract

Each year the proportion of Australians who rent their home increases and, for the first time in generations, there are now as many renters as outright homeowners. Researchers and policy makers, however, know very little about housing conditions within Australia’s rental housing sector due to a lack of systematic, reliable data. In 2020, a collaboration of Australian universities commissioned a survey of tenant households to build a data infrastructure on the household and demographic characteristics, housing quality and conditions in the Australian rental sector. This data infrastructure was designed to be national (representative across all Australian States and Territories), and balanced across key population characteristics. The resultant Australian Rental Housing Conditions Dataset (ARHCD) is a publicly available data infrastructure for researchers and policy makers, providing a basis for national and international research.

## Abstract (summary)

**Table:** 

Measurement(s)	social survey
Technology Type(s)	CATI and online questionnaire
Sample Characteristic - Organism	Homo sapiens
Sample Characteristic - Environment	housing
Sample Characteristic - Location	Australia

Machine-accessible metadata file describing the reported data: 10.6084/m9.figshare.14842914

## Background & Summary

In an Australia dominated by a Great Australian Dream of homeownership, renting has traditionally been regarded as a transitional, rather than a lifetime tenure^1,2^. In recent decades, a decreasing proportion of Australian households have made the transition to home ownership and, recent data shows that, Australians are now more likely to be renters than homeowners without a mortgage^3^. Rental has become Australia’s fastest growing and most diverse tenure with private renting increasing by 64 per cent 2001–2016, twice the rate of household growth^4^. Currently in Australia, 32 per cent of all dwellings are rented, which house almost 3 million households^3^. While the shape of the Australian rental housing market is rapidly changing, we know very little about conditions ‘beyond the front door’ of Australia’s growing rental stock.

Across almost every similar nation, a systematic data infrastructure underlies policy development and monitoring of the rental sector. Currently no parallel data infrastructure exists for Australia. In the absence of such infrastructure, research, government and non-government housing stakeholders have traditionally relied on a relatively piecemeal collection of non-specific, ill-matched, limited sample size data to understand and monitor rental housing related topics. Responding to a clear need for evidence-based policy development based on rental research infrastructure, the Australian Rental Housing Conditions Dataset (ARHCD) described in this paper provides a reliable, robust and contemporary data infrastructure to monitor dwelling conditions in our rental housing stock, based on direct accounts from real-life renters about their home’s condition, in addition it aims to provide researchers and policy stakeholders with a foundational evidence base that they can access, adapt, analyse, and build upon.

The project was funded by the Australian Research Council through the Linkage Infrastructure, Equipment and Facilities (LIEF) grant program, with additional financial support provided by the Australian Housing and Urban Research Institute (AHURI). These two funding sources enabled the collection of large-scale data able to inform a number of different types of analyses focussed on a range of issues.

## Methods

The project commissioned the collection of around 15,000 survey responses from public and private tenants collected between July and August 2020. The survey built upon an earlier survey pilot undertaken in 2016 (described and documented in Baker *et al*.^5^). Representative samples were taken from respondents located across the Australian States and Territories, with sampling relative to population in each State and Territory (described in more depth below). To maximise responses and increase representativeness of the renter population, tenant households were surveyed using a combination of computer-aided telephone interviews (CATIs) and an online survey method.

The research team was responsible for conceiving of the project and securing funding, attaining ethical approvals (granted by The University of Adelaide’s Human Research Ethics Committee (H-2020-069)), and developing survey content and protocols. A third-party agency, EY Sweeney, were contracted to collect the data (including data checking and cleaning during and post survey). On receipt of the final dataset, the research team was responsible for data lodgement and ongoing custodianship.

### Questionnaire development

The questionnaire represents a revision of an earlier housing conditions questionnaire developed by an interdisciplinary team of lead investigators and piloted in 2016^5,6^. The design of the questionnaire draws on existing global exemplars of household and housing panel surveys, for example, the Canadian Housing Survey (https://www.statcan.gc.ca/eng/survey/household/5269), the American Housing Survey (https://www.census.gov/programs-surveys/ahs.html), the New Zealand House Condition Survey (https://www.branz.co.nz/healthy-homes-research/hcs/), the Australian Bureau of Statistics’ (ABS) survey of Housing Mobility and Conditions (https://www.abs.gov.au/ausstats/abs@.nsf/mf/4130.0.55.002), and the Household Income and Labour Dynamics Australia (HILDA) Survey (https://melbourneinstitute.unimelb.edu.au/hilda). The questionnaire was adapted for tenant households with reference to a range of socially- and privately-rented focussed housing surveys and standards, such as the Australian Institute of Health and Welfare’s (AIHW) National Social Housing Survey (https://www.aihw.gov.au/about-our-data/our-data-collections/national-social-housing-survey), the CHOICE Rental Survey (https://www.choice.com.au/money/property/renting/articles/choice-rental-market-report), and New Zealand’s Rental Housing Warranty of Fitness checklist (https://nzrentalwof.co.nz/home). As with every survey, there were limitations to the extent of information collected. Future iterations of the questionnaire instrument may be enhanced by the inclusion of items from other national or international precedents.

The main body of the questionnaire sought information across the characteristics of lease arrangements, dwelling condition and quality, the affordability of rental payments and other financial hardship, the presence of major building problems and maintenance needs, future housing aspirations, and whether the dwelling supported tenants’ security, safety and wellbeing. The survey also collected information on the demographic characteristics, finances, and health of the responding person and other members of their household. The survey was developed in a way that would allow researchers to develop new insights into Australia’s rental housing—its stock and its inhabitants—but also better understand how it intersects with other determinants of wellbeing: income and employment status, gender, presence of a disability in the home, location, and socioeconomic and health status.

Development of the survey occurred during the early stages of the COVID-19 pandemic, which—among widespread social and economic impacts—had a profound effect on the Australian rental sector. Responses to economic hardship included moratoriums on eviction, and rental deferment or reduction arrangements between tenant households and property agents/landlords. To capture the experience of renters through the pandemic, we developed a COVID-19 module—funded by the AHURI under the COVID-19 Agenda Funding Round—that was administered to online respondents in mid-2020. The module was developed in consultation with key policy and research stakeholders, and covered topics such as changes to employment, income and living arrangements, financial hardship, dwelling suitability for working or studying from home, mental health and wellbeing, and anticipated need for future state-based welfare.

The final CATI version of the survey took between 18–19 minutes on average to complete, while the online version (including the COVID-19 module) took 12–13 minutes on average to complete.

### Sampling

Persons over the age of 18 years old were invited to participate in the survey. The sampling strategy was designed to achieve representativeness by population distribution across State and Territory (±0.9 per cent) and where possible, by tenancy type (Table 1), with a natural fallout across gender and age. Public housing tenants (4.2 per cent of the overall population at the last census^7^) were purposefully oversampled to enable stratified analysis of the final data.

**Table 1:** Composition of the final dataset by jurisdiction, tenancy type and survey method.

Sample frame	Responses (count)	Responses (%)
**Location**		
New South Wales	4717	31.4
Victoria	3756	25.0
Queensland	3250	21.7
South Australia	1102	7.3
Western Australia	1410	9.4
Tasmania	313	2.1
Australian Capital Territory	262	1.7
Northern Territory	194	1.3
**Tenancy type**		
Public	1842	12.3
Private	13162	87.7
**Method**		
CATI	1407	9.4
Online	13597	90.6

CATI participants were sourced from a list of renters via a commercial list broker. The list broker was fully compliant with the National Privacy Principals of The Privacy Act 1988, the Commonwealth Government’s Do Not Call Register, as well as the Association of Data-Driven Marketing and Advertising (ADMA) Do Not Mail Register.

Online participants were recruited via an online research panel, involving a pre-recruited group of individuals who have agreed to participate in market research such as online surveys as part of their consent to be on the panel. The research panel recruits panel members using a mix of online and offline methods, which increases the representativeness of their member base against the general population. The panel profile is regularly compared to ABS Census data and recruitment of panellists is targeted to align with ABS data. The panel is committed to protecting the privacy and confidential information of those who register with them and is fully compliant with The Research Society’s Code of Professional Behaviour, Privacy Act 1988 and Spam Act 2003.

### Data collection

Following a pilot phase and a soft launch of the survey in mid to late June 2020, the main fieldwork took place from 30 June to the 22 August. The CATI surveying method yielded fewer interviews than initially anticipated, which was likely influenced by disruptions to normal working and studying arrangements due to the COVID-19 pandemic. The target number of online completes was therefore raised to compensate the shortfall of CATI surveys completed (over 76,700 call attempts and follow-ups were made). We acknowledge that, because data collection occurred during the COVID-19 pandemic, the data reflects a particularly dynamic moment within the Australian rental sector—the research and policy community would no doubt benefit from an ongoing housing conditions monitor.

### Composition of the final dataset

The final dataset includes a total of 15,004 responses from individuals who rent their housing from the government or community sector, and private landlords or real estate agents. Two versions of the questionnaire were administered; the CATI version, which excluded the COVID-19 module (n = 1,407), and the online version, which included the COVID-19 module (n = 13,597). The initial pilot launch began with the CATI surveys, which included n = 17 completed interviews, which have been included in the final dataset. Minor adjustments were made to the survey questions and flow following the pilot phase.

The final questionnaire involved presenting CATI respondents with approximately 41 questions and online respondents with approximately 59 questions; some variances were noted in the number of questions asked based on participants’ responses. Items covering housing dissatisfaction, location satisfaction, and the impacts of rising housing costs were removed during the pilot and soft launch of the survey for clarity and to reduce survey length.

In accordance with the ethical approval, all respondents gave their informed consent at the start of the survey. Their consent was attained under the conditions that the data were de-identified prior to analysis or sharing, that the data were securely stored, and that the data was to be used for research and policy purposes only.

Table 1 presents the final sample by location (State or Territory), tenancy type (public versus private), and survey method (CATI versus online). The composition of the final sample reflects the sampling strategy, with slight under-representation of public housing tenant respondents in the Northern Territory, and slight over-representation of Victorian and South Australian renters.

## Data Records

The ARHCD is lodged with the Australian Data Archive^8^. Both sensitive and non-sensitive versions are accessible via 10.26193/IBL7PZ upon registration and request. The details of the two versions (sensitive and non-sensitive) of the ARHCD are provided Table 2. The data are as received from EY Sweeney with the exception of the removal of the postcode variable from the non-sensitive version of the dataset. No other manipulation has occurred.

**Table 2:** Details of the ARHCD versions lodged with the Australian Data Archive.

File name	File type	Notes
02_AHCD_Non_Sensitive_Data_File_001469	.sav; .sas; .dta; .sas7bdat	Final *non-sensitive* version of the dataset without postcode variable. Access upon registration and request, managed by ADA.
02_AHCD_ Sensitive_Data_File_001469	.sav; .sas; .dta; .sas7bdat	Final *sensitive* version of the dataset containing the postcode variable. Access upon registration and request, managed by ADA.

The final versions of the two surveys and data dictionaries are also available via the ADA Dataverse webpage^8^.

## Technical Validation

### Data checking and cleaning protocols

All data processing requirements were conducted in-house by EY Sweeney. The data cleaning and validation process consisted of:

- Verification of the automated checking of data, including logic checks of the data against known profiles or pre-defined ranges and checks for any empty cells;
- A review to ensure correct labelling of variables and values;
- Verification of response inconsistencies;
- Validation to ensure the data was captured in the required format and with only permissible values; and
- De-identification of data, including verbatim responses, to protect the privacy and confidentiality of individuals who participated in the survey.

The data were checked and cleaned during the fieldwork phase, which continued until after the end of the data collection period. While the majority of potential issues with the data was addressed via logic checks at the programming stage (e.g. restricting the types of data that could be entered), a range of data checks were also completed upon commencement of fieldwork, including after the pilot survey. At the end of fieldwork period, the data was exported to SPSS for final cleaning and validation, where a final check was conducted.

### Representativeness

The ARHCD, including the COVID-19 module, achieved a reasonably balanced sample, where quotas were used to achieve representativeness by population distribution across State and Territory and where possible, by tenancy type (based on a question relating to landlord type), with a natural fallout. Respondents living in social housing (public and community sectors) were purposely over-sampled to allow for adequate sample sizes for stratified analysis. Online-only Table 1 presents a comparison of select demographic characteristics of the ARHCD sample with the most recent Australian Census collected in 2016.

## Usage Notes

To download the ARHCD, users are required to register with the ADA and log a request to access the data files. On request, users are required to provide the following information:

- Name
- Email
- Institution or Organisation
- Position
- What is your primary intended use of this data?
- If other uses, please indicate what other intended use you have for this data
- Please provide a brief abstract outlining the intended use of this data for your project
- Please detail any sources of funding supporting this research

The request approval process is managed by ADA.

To maximise the utility of the ARHCD for housing and urban research, users may consider adding functionality to the dataset by geo-coding responses (postcodes available in the sensitive version only), or by formulating design and/or non-response weights. All open-ended questions or responses to “Other, please specify” are include verbatim.

The data are not intended for commercial use.

## Data Availability

No post hoc manipulation of the data has occurred. The ARHCD files accessible via the ADA are available in .sav; .sas; .dta; and .sas7bdat formats^8^.

## Online-only Table

**Online-only Table 1:** Comparison of the age, sex, landlord type and household income of the Australian Rental Housing Conditions Dataest (ARHCD) renter sample with the 2016 ABS Census, by State or Territory.

Variable	Category (source)	Australian State or Territory								
		NSW	VIC	QLD	SA	WA	TAS	ACT	NT	ALL
Sex*/Gender	%Female (ARHCD)	57	57.7	63.9	56.5	62.5	57.5	53.8	58.7	59.1
	%Female (Renters in 2016 Census)	50.6	50.7	51.1	50.9	50.5	51.9	49.8	50.2	50.7
Age	18 to 29 years (ARHCD)	26.3	30.4	26.3	24.3	29.5	30.7	38.3	38.8	27.9
	18 to 29 years (Renters in 2016 Census)	31.8	35.9	33.8	32.1	33.2	32.2	32.4	38.0	33.5
	30 to 49 years (ARHCD)	51.8	50.5	45.5	46.7	47.2	42.1	45.8	42.6	48.9
	30 to 49 years (Renters in 2016 Census)	42.6	42.3	40.4	39.1	42.5	37.0	44.7	42.9	41.7
	50 to 64 years (ARHCD)	16.0	14.6	21.1	20.6	18.1	22.3	13.8	10.1	17.3
	50 to 64 years (Renters in 2016 Census)	16.6	14.3	16.8	17.6	15.8	18.4	16.8	13.0	16.1
	65 years or over (ARHCD)	5.9	4.4	7.1	8.4	5.2	4.9	2.0	8.5	5.8
	65 years or over (Renters in 2016 Census)	9.0	7.5	9.0	11.2	8.5	12.3	6.1	6.2	8.7
Landlord type	A real estate agent (ARHCD)	73.5	77.3	71.4	58.7	67.4	54.9	62.9	58.6	71.6
	A real estate agent (Renters in 2016 Census)	68.8	70.6	65.8	50.5	52.7	45.0	32.1	52.5	64.5
	A State or Territory housing authority (ARHCD)	6.6	6.5	6.9	12.6	8.2	14.8	18.5	13.2	7.7
	A State or Territory housing authority (Renters in 2016 Census)	10.9	7.6	8.7	15.2	11.8	16.1	34.3	17.2	10.6
	Someone not in the same household (ARHCD)	11.3	10.8	15.1	18.2	17.1	20.9	14.5	22.4	13.4
	Someone not in the same household (Renters in 2016 Census)	17.3	17.3	18.8	26.0	26.1	31.1	13.8	22.6	19.4
	A community housing provider (ARHCD)	1.8	1.7	2.4	2.4	2.2	1.7	2.0	2.9	2.0
	Housing co-operative/community/church group (Renters in 2016 Census)	1.7	1.2	1.2	2.9	1.4	2.6	6.1	1.3	1.6
	Other (ARHCD)	6.8	3.8	4.3	8.1	5.0	7.7	2.0	2.9	5.3
	Other (Renters in 2016 Census)	1.2	0.7	2.2	1.5	2.3	0.9	7.8	3.9	1.6
Household income	Less than $31,000 (ARHCD)	15.4	16.1	18.6	23.7	18.8	30.7	12.2	16.2	17.5
	less than $33,800 (Renters in 2016 Census)	18.1	19.5	17.2	25.9	18.5	29.1	13.6	14.4	18.8
	$31,000-$59,000 (ARHCD)	18.6	18.5	23.6	24.6	20.1	20.0	18.3	24.0	20.3
	$33,800-$64,000 (Renters in 2016 Census)	24.2	25.3	27.5	31.2	23.5	34.8	19.8	18.1	25.6
	$59,001-$90,000 (ARHCD)	18.4	23.4	22.4	20.9	23.3	23.9	20.0	19.8	21.3
	$65,000-$90,999 (Renters in 2016 Census)	16.3	17.2	19.1	17.3	16.2	16.4	16.4	16.0	17.2
	$90,001-$125,000 (ARHCD)	18.2	19.0	16.7	16.0	16.0	16.4	18.3	14.4	17.6
	$91,00-$129,999 (Renters in 2016 Census)	18.7	18.8	19.6	14.9	19.3	12.2	21.2	22.2	18.7
	$125,001-$150,000 (ARHCD)	11.7	9.7	8.0	7.9	9.7	3.2	9.6	10.2	9.7
	$130,000-$155,999 (Renters in 2016 Census)	7.1	6.7	6.6	4.4	7.2	3.2	8.2	9.6	6.7
	$150,001-$175,000 (ARHCD)	6.2	4.7	4.1	3.4	4.2	2.1	7.4	6.0	4.9
	$156,000-$181,999 (Renters in 2016 Census)	4.5	3.9	3.6	2.2	4.3	1.5	5.8	5.9	4.0
	$175,001-$200,000 (ARHCD)	5.4	4.0	3.3	2.2	3.4	2.1	9.6	6.0	4.2
	$182,000-$207,999 (Renters in 2016 Census)	4.1	3.4	2.7	1.8	4.4	1.3	5.6	5.5	3.5
	Over $200,000 (ARHCD)	6.1	4.5	3.2	1.3	4.6	1.4	4.8	3.6	4.4
	Over $208,000 (Renters in 2016 Census)	7.0	5.1	3.8	2.4	6.6	1.5	9.4	8.2	5.4

*The 2016 ABS Census only collected limited data on gender, comparisons here have been made by sex (the 49 ARHCD respondents nominating “Other” gender were excluded from the comparison above).
